# Personal

**Published:** 1910-11

**Authors:** 


### PERSONAL

DR. J. HILTON WATERMAN, formerly of Buffalo, now of New York, has removed from 50 West Fifty-First Street, to 205 West Fifty-Seventh Street. Hours: 10 to 11 a. m., 5 to 6 p. m. Telephone: Columbus 8391.

DR. FRANCIS E. FRONCZAK, health commissioner, of Buffalo, will deliver an address on the subject of hygiene before a health conference on November 22, 1910, at the Johns Hopkins University, Baltimore, Md., according to a recent announcement.

DR. ALBRECHT KOSSEL, professor of physiology at Heidelberg University, has been awarded the Nobel prize for medicine. Professor Kossel is in his 58th year. He is a native of Bostock and he studied medicine there and in Strassburg. He has done much pioneer work in the artificial production of organic material.

